# Supplementary material for: Diverse modes of synaptic signaling, regulation, and plasticity distinguish two classes of C. elegans glutamatergic neurons
Source: eLife. 2017 Nov 21;6:e31234. doi: 10.7554/eLife.31234 (PMC5705214; doi:10.7554/eLife.31234)
Supplement: Supplementary file 1. [file elife-31234-supp1.docx]

***C. elegans* strains**

| CX14174 | *kyEx4435: AWC^ON^ VGLUT-pH Line*:  s*tr-2:eat-4::pHluorin (*30 ng/ul); *unc-122:dsRed* co-injection marker (15 ng/ul). |
| --- | --- |
| CX16644 | *kyIs623*: UV-integrated *kyEx4435 AWC^ON^ VGLUT-pH Line.*  Backcrossed to N2 11x. |
| CX16921 | *kyIs673*: *ASH VGLUT-pH Line*  *sra-6:eat-4::pHluorin (50 ng/ul); unc-122:dsRed* co-injection marker (20 ng/ul). UV-integrated, backcrossed to N2 8x |
| CX15571 | *kyEx5236*: *AWC^ON^ syGCaMP3*  *str-2:sng-1::GCaMP3*; *unc-122:dsRed* co-injection marker (15 ng/ul). |
| CX10979 | *kyEx2865 ASH GCaMP3*  *sra-6:GCaMP3 (100 ng/ul); unc-122:GFP (10 ng/ul)* |
| CX17105 | *kyIs623; kyEx5969: AWC^ON^ Tetanus toxin*  *str-2:Tetx:sl2:mCherry (30 ng/ul); unc-122:GFP (20 ng/ul)* |
| CX13914 | *KyEx4275: AWC^ON^ GCaMP5A*  *str-2:GCaMP5A D380Y (50ng/ul); unc-122::dsRed (10ng/ul)* |
| CX17068 | *kyIs673; KyEx1661 ASH Tetanus toxin*  *sra-6:Tetx:sl2:mCherry (50 ng/ul); elt-2:NLS-GFP* |
| CX16954 | *unc-64(e246); kyIs623* |
| CX17076 | *unc-64(e246); kyIs673* |
| CX17071 | *ric-4(md1088); kyIs623* |
| CX17104 | *ric-4(md1088); kyIs673* |
| CX15568 | *unc-13(s69); kyEx4435* |
| CX17072 | *unc-13(s69); kyIs673* |
| CX15416 | *unc-18(e234); kyEx4435* |
| CX17075 | *unc-18(e234); kyIs673* |
| CX16451 | *kyEx5608: AWC^ON^ cyto-pH*  *str-2:pHluorin (30 ng/ul); unc-122:dsRed (20 ng/ul)* |
| CX16963 | *kyEx5901: ASH cyto-pH*  *sra-6:pHluorin (100 ng/ul); unc-122:dsRed (20 ng/ul)* |
| CX16491 | *unc-18(e234); kyEx5608* |
| CX16494 | *unc-13(s69); kyEx5608* |

| CX17080 | *unc-10(md1117); kyIs623* |
| --- | --- |
| CX17074 | *unc-10(md1117); kyIs673* |
| CX15850 | *cpx-1(ok1552); kyIs623* |
| CX17069 | *cpx-1(ok1552); kyIs673* |

| CX16967 | *kyEx5904: ASH CD4-pH*  *sra-6:pHluorin::CD4 (100 ng/ul); unc-122:dsRed (20 ng/ul)* |
| --- | --- |
| CX17096 | *unc-11(e47); kyIs623* |
| CX17109 | *unc-11(e47); kyIs673* |
| CX16165 | *pkc-1(nj1); kyEx4275* |
| CX16318 | *pkc-1(nj1); kyEx5236* |
| CX15609 | *pkc-1(nj1); kyEx4435* |
| CX15848 | *pkc-1(nj1); kyIs623* |
| CX15849 | *pkc-1(nj1); kyIs623; kyEx5354*  *kyEx5354 =str-2:pkc-1(cDNA):sl2:mCherry (30ng/ul) ; Co-injection marker =elt-2:mCherry (5ng/ul).* |
| CX16950 | *pkc-1(nj1); kyIs673* |

| CX16645 | *pkc-1(nj3); kyIs623* |
| --- | --- |

|  | *pkc-1(ok563); kyIs623* |
| --- | --- |
| CX16952 | *pkc-1(nu488); kyIs623* |
