# Supplementary material for: Diverse modes of synaptic signaling, regulation, and plasticity distinguish two classes of C. elegans glutamatergic neurons
Source: eLife. 2017 Nov 21;6:e31234. doi: 10.7554/eLife.31234 (PMC5705214; doi:10.7554/eLife.31234)
Supplement: Supplementary file 2. [file elife-31234-supp2.docx]

**Molecular identity of mutations**

| *unc-64(e246)* | Ala 🡪 Val missense mutation at codon 248 in the conserved H3 domain. RRID:WB-STRAIN:CB246 |
| --- | --- |
| *ric-4(md1088)* | Staunton et al., J. Neuro 2001; mutation indicated as hypomorphic, molecular lesion not described. RRID:WB-STRAIN:RM956 |
| *unc-13(s69)* | 5-bp deletion in exon 21, frameshift followed by early translation termination. Predicted null for LR and MR isoforms. RRID:WB-STRAIN:BC168 |
| *unc-18(e234)* | C🡪T mutation leading to translation termination in exon 4. Predicted null mutation. RRID:WB-STRAIN:CB234 |
| *unc-11(e47)* | Indel covering exons 1 and 2, resulting in frameshift and early translation termination. Predicted null mutation. RRID:WB-STRAIN:CB47 |
| *unc-10(md1117)* | 8601 bp deletion spanning the entire gene. Null mutation. RRID:WB-STRAIN:NM1657 |
| *cpx-1(ok1552)* | 1274 bp deletion that removes one of two exons. Predicted null mutation. RRID:WB-STRAIN:RB1367 |
| *pkc-1(nj1)* | Asp 🡪 Asn missense mutation in the catalytic loop of the kinase domain, affecting codon 502 (position numbering in a isoform). RRID:WB-STRAIN:IK105 |
| *pkc-1(nj3)* | G 🡪 A mutation leading to translation termination of all isoforms before the kinase domain, at W218 (a isoform). RRID:WB-STRAIN:IK130 |
| *pkc-1(nu488)* | Nonsense mutation (W643Stop, a isoform)) leading to translational termination within the kinase domain of all isoforms. RRID:WB-STRAIN:KP2342 |
| *pkc-1(ok563)* | 1.3 kb deletion, predicted to disrupt a and c isoforms before the kinase domain. RRID:WB-STRAIN:RB781 |
