# Supplementary material for: Diverse modes of synaptic signaling, regulation, and plasticity distinguish two classes of C. elegans glutamatergic neurons
Source: eLife. 2017 Nov 21;6:e31234. doi: 10.7554/eLife.31234 (PMC5705214; doi:10.7554/eLife.31234)
Supplement: Supplementary file 3. [file elife-31234-supp3.docx]

**Supplementary File 3A**

**Supplementary File 3B**

**Supplementary File 3C**
